# Design of the New Life(style) study: a randomised controlled trial to optimise maternal weight development during pregnancy. [ISRCTN85313483]

**DOI:** 10.1186/1471-2458-6-168

**Published:** 2006-06-26

**Authors:** Ellen Althuizen, Mireille NM van Poppel, Jacob C Seidell, Carla van der Wijden, Willem van Mechelen

**Affiliations:** 1 Department of Public and Occupational Health, EMGO-Institute, VU University Medical Center, Amsterdam, The Netherlands; 2 Research Center Physical Activity, Work and Health, TNO-VU, Amsterdam and Hoofddorp, The Netherlands; 3 Department of Nutrition and Health, Faculty of Earth and Life Sciences, VU University Amsterdam, The Netherlands; 4 Department of Obstetrics and Gyneacology, Medical Center Amstelland, Amstelveen, The Netherlands

## Abstract

**Background:**

Preventing excessive weight gain during pregnancy is potentially important in the prevention of overweight and obesity among women of childbearing age. However, few intervention studies aiming at weight management during pregnancy have been performed and most of these interventions were not as successful as expected. In this paper the design of the New Life(style) study is described as well as the content of the individually tailored intervention program, which focuses on controlling weight development during pregnancy.

**Methods:**

The effectiveness of the New Life(style) intervention program versus usual care by midwives is evaluated in a randomised controlled trial. Women who expect their first child and visit one of the participating midwifery practices are included. The intervention is standardised in a protocol and executed by trained counsellors with the women who are randomised in the intervention group. During 5 sessions – at 18, 22, 30 and 36 weeks of pregnancy and at 8 weeks postpartum – individual weight gain is discussed in relation to weight gain guidelines for pregnant women of the American Institute of Medicine. Counsellors coach the women to maintain or optimise a healthy lifestyle, in a period of drastic physical and mental changes. Data is collected at 15, 25, 35 weeks of pregnancy and at 6, 26, and 52 weeks after delivery. Primary outcome measures are body weight, BMI, and skinfold thickness. Secondary outcome measures include physical activity, nutrition and blood levels of factors that are associated with energy homeostasis.

**Discussion:**

Results of the current RCT will improve the knowledge of determinants of weight gain during pregnancy, weight retention after childbirth and of the effectiveness of the intervention program that is described. Caregivers and researchers in the field of health promotion are offered more insight in specific elements of the New Life(style) intervention program.

## Background

Over 1.1 billion adults and 10% of children worldwide are now classified as overweight or obese [1]. Because of a substantial adverse impact on health and well-being [2], stabilising or decreasing the number of people with overweight is addressed as a target of many regional and national health programs in developed countries [3]. As different life events can trigger a shift in the energy balance resulting in positive weight changes and a higher risk of developing overweight, these moments are thought to be the moments when people will benefit most from health-promoting activities. Therefore, subgroups such as children who are starting school, retired or sick people, are often the target group of health promoting programs [4].

In this manuscript we elaborate on the New Life(style) project. In this study the focus is on pregnant women, because pregnancy has shown to be an independent risk factor for developing overweight [5-8]. Although the American Institute of Medicine (IOM) developed evidence based guidelines for weight development during pregnancy in 1990, still 20% to 40% of women gain more weight than advised by the IOM [9]. Weight gain during pregnancy is considered to be the most important determinant of postpartum weight retention [10,11]. Studies describe an average weight retention of 2–3 kg, 6 to 12 months after delivery and losing this "extra" weight after having given birth proves to be difficult [12,13]. For this reason, preventing excessive weight gain during pregnancy is important in the prevention of overweight and obesity among women of childbearing age.

Although it has been recognised that pregnancy increases the risk of developing overweight later in life, only few intervention studies aiming at weight management during pregnancy have been performed. Moreover, most of these interventions were not as successful as hoped. Polley et al. [14] were the first to evaluate the effect of a stepped-care, behavioural intervention. They found that the intervention was effective in reducing the incidence of excessive weight gain in normal-weight women, but not in overweight women. No significant effects were found with regard to total weight gain or net weight retention. An intervention study consisting of regular, individual diet counselling, physical activity sessions and other activities related to nutrition was conducted by Gray-Donald et al. [15]. They found only a minor effect on diet, but no effect on weight gain or physical activity. Finally, Olson et al. [16] reported a reduced risk of excessive gestational weight gain in a low-income subgroup of women only. Those women were monitored by health care providers and received patient education by mail.

An intervention program that will be effective for every individual is unrealistic, but it is clear that the need for effective strategies concerning weight management during pregnancy will remain. For this reason, an individually tailored intervention was developed for the study that is discussed in this paper; i.e. the New Life(style) intervention program. The main focus of this intervention is to help pregnant women to gain weight within IOM-guideline limits by 1) informing them on these guidelines, but also on physical activity and nutritional guidelines for pregnant women; 2) reviewing individual lifestyle and 3) giving support to optimise individual lifestyle during pregnancy.

The present paper reports on the methodological design of the New Life(style) study as well as the content of the intervention program. Publishing the design and rationale of an RCT before results are available has important benefits [17]. It is an opportunity to elaborate on the content and background of an intervention program and to consider the methodological quality of the study critically, irrespective of the results. As a consequence, caregivers and researchers in the field of health promotion are offered more insight in specific elements of intervention programs.

## Methods

### Study design

**Figure 1:**
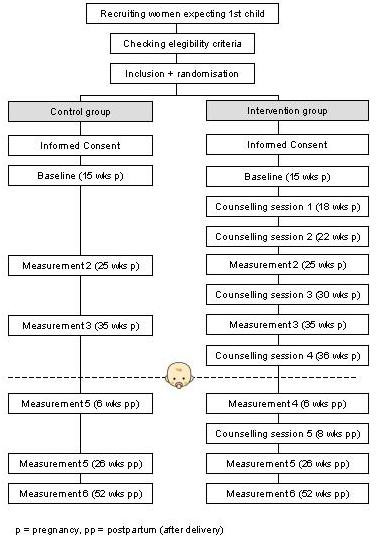
Study design.

The study is designed as a randomised controlled trial to evaluate the effectiveness of a lifestyle intervention program with the main focus on weight development, physical activity and nutrition habits during pregnancy. The design is presented in Figure 1. The Medical Ethics Committee of VU University Medical Centre has approved the study design, protocols and informed consent procedure. Participating women are assigned at random to the control or to the intervention group. They are studied during a period of 18 months: from 15 weeks of pregnancy until one year after delivery.

### Setting

This trial is carried out in eight midwifery practices in Dutch towns with 23,000–735,000 inhabitants. The midwives working in these practices ask the women to participate in the study from February 2005 until May 2006. By selecting practices in different urbanised agglomerations we aim to include a selection of midwives and participants that is representative of these subgroups in the Western part of the Netherlands. Participation in the study is voluntary.

### Midwifery in the Netherlands

In the Netherlands midwives are independent paramedical practitioners, qualified to provide full maternity care to all women whose pregnancy and childbirth are uncomplicated. The primary task of the Dutch midwife is to follow closely the health status of the pregnant woman and unborn child [18]. The first appointment at the midwifery practice takes place between the 9^th ^and 12^th ^week of gestation. Pregnant women visit the midwives 11–13 times during their pregnancy for about 15 minutes each time. Eventually, thirty percent of births occur at home, assisted by these midwives; seventy percent of pregnant women deliver in hospitals [19]. Because of the frequent contacts throughout pregnancy and because of their expertise, midwives are considered to be important information providers of pregnant women, especially for women without children who visit the midwife for the first time in their lives. For some women midwives have a corrective, reassuring and reinforcing role.

### Rationale for using midwifery practices as the research setting

The unique platform of midwifes that exists in the Netherlands is very appropriate for conducting this trial with pregnant women as the women are recruited for participation by esteemed health care providers, and not via impersonal invitation letters. By individually addressing the women by the midwives we hope to optimise the inclusion of participants. Another advantage of using midwifery practices as the research setting is that the appointments of participants with their midwives can be combined with appointments for research purposes. Extra visits of participants to a research centre are not necessary, reducing the time consuming consequences of an extensive research program with several succeeding measurement moments.

### Recruitment of midwifery practices

Twenty five midwifery practices were approached and invited to participate in this study. Some practices were recruited via personal work-related contacts and others via the phone-book. All practices are within an area of 60 kilometres from the VU University Medical Center in Amsterdam and are accessible by public transport; an accessibility criterion. Furthermore, participating practices have to provide a room once a week, for measurements and the counselling sessions that constitute the intervention program.

The approached practices received a brochure about the study and were called one week later by the research team, to ask for participation. Nine of the twenty five practices were too busy to participate in a study, three practices were not interested in the subject of weight gain during pregnancy, three practices had no spare room, and two already participated in another study.

During the study, there will be no data exchange between researchers and midwives (e.g. group allocation, anthropometric data, content of counselling sessions). Midwives are instructed to refer participants with questions about the study to the research team and to provide usual care to all their clients, irrespective of their participation or randomisation status.

### Study population

#### Sample size

The aim is to include 275 participants in the study. A power analysis has been based on the effects of the intervention program on BMI of participants in the intervention group. In order to be able to detect a clinically relevant difference in BMI of 0.8 kg/m^2 ^at six months postpartum between the intervention and control group, 220 participants are needed, 110 participants in each group. These numbers are based on a power (1-B) of 0.80, a significance level of 5% (two-sided) and a standard deviation of 2.0 kg/m^2^. When a drop out of 20% is taken into account, which is normal for similar research with 1 year follow up, 275 women are needed for this trial.

### Selection of participants

Subjects participating in the study are recruited by participating midwives. During the intake, midwives invite women who are expecting their first child to participate. They introduce the study to the new client and give the women who are interested a brochure about the trial, to be read at home. After having received consent to do so, midwives write the name, telephone number, body weight, and expected date of delivery of interested women on a reply coupon, and send this coupon to the research team. Subsequently, a research assistant calls interested women within two weeks after the introduction to the study by her midwife. During this conversation the aim and implications of the study are explained again and the eligibility criteria are checked. Women are eligible for participation when they are:

1) expecting their first child;

2) able to read, write and speak Dutch;

3) within their first 14 weeks of pregnancy.

When a woman meets these inclusion criteria and wishes to join the study, she is included. After inclusion, an appointment is made for the baseline measurement at the midwifery practice. Informed consent forms will be obtained from each participant.

This study focuses on women who are expecting their first child, and who will deliver their first child during the term of the study. By excluding pregnant women who already have children, the study population is expected to be more homogeneous. Mothers that are expecting their second or third child, have already experienced at least one pregnancy and the weight change related to that pregnancy. Based on the experiences in their previous pregnancy, these women already have specific expectations and attitudes concerning weight changes and lifestyle during pregnancy. Besides, the daily situation of women with children is different. Because of the transition from a household of adults to a family life, their lifestyle has already been changed. So, focusing on women without children limits disturbing effects of parity on outcome measures.

### Non-participants

Midwives are asked to fill in anonymous reply coupons for every woman who is not interested to participate in the study; including weeks of pregnancy, body weight and the reason for not participating. Women who decide not to participate after reading the information brochure at home, are asked to return a similar coupon. Characteristics of this group of non-participating women will be compared to those of women who do participate in the study.

### Randomisation

A computerized random number generator draws up an allocation schedule prestratified for midwifery practice; within practices respondents are allocated to the intervention group or to the control group at random. During the first telephone call with the research assistants – 2 weeks after the first appointment with the midwife; at 10–14 weeks of pregnancy – participants are informed about which group they are assigned to.

### Blinding

All anthropometric outcome measures are assessed by independent examiners unaware of group allocation. Participants cannot be blinded for the intervention, but are asked not to reveal information about their treatment to the examiners. The key of coding concerning group assignment is only known by the programmer of the database that is used during the study.

### Intervention

The New life(style) intervention program consists of 5 individual counselling modules together with a general information brochure. In the modules the counsellors discuss with the women allocated to the intervention group, how to control their weight during and after pregnancy, and how to maintain or optimize a healthy lifestyle in a period of drastic physical and mental changes. Women go through these counselling sessions in 30 weeks; they have appointments with their counsellor at 18, 22, 30 and 36 weeks of pregnancy and at 8 weeks postpartum. The counsellors, in charge of these counselling sessions, are members of the research team (see Counsellors). They are permanently linked to a specific midwifery practice. In this way, all women in the intervention group are assigned to a personal counsellor, facilitating the contact between participant and counsellor. Each counselling session takes 15 minutes, but the first session takes approximately half an hour, to explain the aim of the study and the intervention. The content of the first module is generalised and summarised in an information brochure, which the participants receive after the first counselling session of the intervention program.

### Rationale of the intervention program

During the New Life(style) program pregnant women in the intervention group receive individually tailored advice. The personal counsellor is a source of information, making the participants aware of the fact that they themselves can influence their weight during pregnancy, but also after delivery. By providing regular guidance following the principles of Problem Solving Treatment for primary care (PST-pc) the counsellor is also a supervisor and coach for the pregnant women. PST is an evaluated, practical psychological intervention method for helping people to gain a sense of control over their difficulties [20]. PST consists on the following elements: 1) increasing one's knowledge; 2) enhancing one's capability to define behaviors in need of change; 3) learning to seek solutions for problems or to set goals to improve or preserve healthy behavior; and 4) emphasizing positive experiences to increase self-confidence [21].

### Content of the counselling sessions

Each counselling session is structured in a uniform way (see Table 1). During the first visit the counsellor talks about weight gain during pregnancy and the distribution of this 'extra' weight among different tissues. After this, the IOM guidelines are introduced and the dependence of healthy weight gain during pregnancy on pre-pregnancy body mass index is discussed. Weight and height is assessed and pre-pregnancy BMI is calculated, after asking for pre-pregnancy weight, in order to categorize women in pre-pregnancy BMI-groups: underweight, normal weight, overweight or obese.

### Weight gain charts

**Figure 2:**
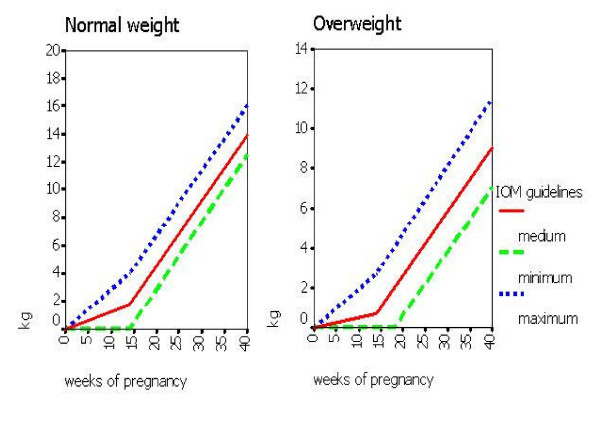
Weight gain charts for normal and overweight women (IOM).

For each pre-pregnancy BMI-group the minimum, mean and maximum weight gain recommendations of the IOM are incorporated in weight gain charts as lines that reflect the accumulative amount of kilograms a women can gain per week, until 40 weeks of gestational age. The women receive a chart, depending on the BMI-group they belong to. On this individual chart weight changes are plotted every time participants of the intervention group visit the counsellor (see Figure 2). Women whose weight changes are within appropriate ranges are informed that they are gaining the expected amount of weight; those gaining too little or those who gain more than recommended are told so.

### Lifestyle

During the remaining time of the first session, but also during follow up sessions, weight gain patterns of the women in relation to their lifestyle is discussed: "how to eat healthy and how to optimise physical activity in order to be able to be in charge of your own weight". Focussing on the relation between energy intake and energy expenditure is an important component of the intervention program. The level of physical activity is assessed by means of the shortened version of the IPAQ [22]. A global assessment of energy intake is done by means of a short questionnaire of the Dutch Nutrition Centre [23] so that the counsellor can give immediate feedback on individual, daily physical activity and nutrition.

Recent studies argue the need for pregnant women to remain physically active during pregnancy, in the absence of contra-indications, because of the beneficial health effects for both mother and child [24]. The information and feedback that the counsellors provide with regard to physical activity are therefore based on the widely accepted recommendations of the American Centers for Disease Control and Prevention, which promote 30 minutes of at least moderate intensity activity on 5 or all days of the week [25]. Guidelines for pregnant women of the Dutch Nutrition Centre constitute the basis of the nutritional part of the counselling sessions [23].

### Goals

The primary goals of the intervention program with regard to physical activity are to provide information and guidance to optimise daily physical activity during pregnancy and to take away common misconceptions about physical activity and exercise during pregnancy (e.g. "exercising is dangerous", "a lot of rest is good for pregnant women"). To accomplish maintenance of a healthy physical activity pattern or to optimise individual physical activity when women don't meet physical activity guidelines; safe, enjoyable, accessible and feasible physical activities are discussed on the individual level. To prevent injuries because of being untrained, previously sedentary women are advised to increase physical activity in small steps and with regard to activities of moderate intensity and low impact, such as cycling instead of taking a bus, or walking during lunch breaks. The wellbeing of the unborn child is always taken into account; counsellors will talk about possible contra-indications for physical activity. The dietary intervention focuses on optimising energy-intake, adjusting energy-intake to physical activity and to take away misconceptions about nutritional requirements during pregnancy (e.g "the need to eat for two" [26]). Special attention is given to decreasing intake of high-fat foods, such as fast food items and sugar-containing soft drinks.

Subsequently, the counsellor makes a plan with the women who don't meet the weight gain, physical activity or dietary guidelines, but also with those who foresee difficulties later in pregnancy. In this plan, goals with regard to physical activity and/or nutrition are incorporated as explicitly as possible, in order to make it easier to achieve them (e.g. by describing the type, frequency, intensity and duration of planned physical activities). Counsellors complete a form after each session in which they register weight gain pattern, the content of each conversation and personal goals that have been set. During subsequent visits, the content of the previous session is evaluated and, if necessary, plans are adjusted.

### Counselling session 5

The fifth and last counselling session, at 8 weeks postpartum, is done by phone. The focus of this session is mainly on recovery from delivery, breast feeding and how to take care of the new baby, in combination with regular physical activity and/or healthy nutritional patterns.

### Counsellors

Two counsellors are responsible for the execution of all counselling sessions with participants in the intervention group. They have a background in physical activity or remedial education and were trained before the start of the study to provide individual feedback on the modules, according to the earlier described standardized counselling protocol. After the counsellors studied related literature and the protocol, the training was performed with ten pregnant women. The conversations with these ten pregnant women allowed the counsellors to become acquainted with the protocol and the target group. The conversations were taped so that they could be studied by the research team, consisting of an epidemiologist, a health scientist, a gynaecologist and the two counsellors. It was checked whether the counsellors adhered to the protocol and problems which the counsellors ran across were discussed. During the study all sessions are taped as well, and regular follow-up sessions with the research team are organised in order to discuss taped conversations.

### Co-interventions and compliance

The participating midwives are asked not to take part in other research programs in order to prevent possible disturbing effects from co-interventions. Compliance to the intervention programme is assessed by registering the number of treatment sessions attended by the participants. Furthermore, the content of each session is registered by the counsellors in individual files of the participants in the intervention group.

### Measurements

Participants in both the intervention and control group are invited for six measurement appointments in the period during and after pregnancy. The baseline measurement is at 15 weeks of pregnancy. Data are collected at: 15, 25, and 35 weeks of pregnancy and at 8, 26, and 52 weeks after delivery. At these occasions the research assistants perform anthropometrical measurements in all midwifery practices and register the primary outcome measures. Blood samples are collected in five of the eight participating practices by trained assistants; in the other three practices the assistants are not schooled for intravenous punctures and consequently no blood samples can be collected. Each assistant is permanently linked to one or more practices, so that participants are measured by the same person at each occasion. All participants receive questionnaires by ordinary mail one week before each measurement appointment. They are asked to complete the questionnaires and hand these in when they visit the research assistant for a measurement appointment. The assistant personally checks the questionnaires for missing data. The following measures are assessed:

### Primary outcome measures

#### Body weight and BMI

Body height is measured on bare feet by means of a wall-mounted height scale (SECA 206), with an accuracy of 0.1 cm. Calibrated electronic scales (SECA 888) are used to determine body weight of the participants in underwear and pants, with an accuracy of 0.1 kg. Both weight and height is measured twice, and the mean value of the two measurements is computed and used to calculate individual body mass index (BMI, kg/m^2^) at 26 and 52 weeks after pregnancy. Pre-pregnancy BMI is based on self reported pre-pregnancy weight.

### Skinfold thickness and percentage body fat

A Harpenden calliper is used to assess skinfold thickness of the following four skinfolds: biceps, triceps, subscapular and suprailiac, according to the method described by Weiner and Lourie [27]. All skinfolds are assessed twice. A mean value of the two is computed. In case the two measurements of a skin fold differ more than 1.0 mm, the skin fold is measured a third time and the mean value of the three values is calculated.

### Secondary outcome measures

#### Physical activity and nutrition

Physical activity is assessed with the Short Questionnaire to Assess Health enhancing physical activity (SQUASH). This questionnaire has been validated in a population of Dutch employees [28]. At 15 and 35 weeks of pregnancy and 26 weeks after delivery all participants wear an accelerometer for 4 consecutive days (actigraph, GT7164). Accelerometer data are used as additional, objective data on physical activity. With regard to nutrition, questions have been developed for the purpose of this study, concerning the amount of times per week that participants have breakfast, lunch and dinner. Also the frequency of eating snacks is assessed. The validated Dutch Eating Behaviour Questionnaire (DEBQ) is used to identify nutrition related problems such as restrained, emotional, and external eating [29].

### Blood samples

In order to study the influence of lifestyle factors on energy homeostasis and weight gain, blood samples are taken intravenously from a subgroup of participants during each measurement. The midwives also take a blood sample from the umbilical cord. Blood is processed and stored at the laboratory of the VU University Medical Centre, so that – after selecting subgroups of normal and high gainers – blood levels of leptin, ghrelin, fasting insulin/glucose, insulin growth factor 1, insulin growth factor binding protein 1 and 3, and cortisol can be measured.

By means of the questionnaires, data are also collected on: perceived health, stage of change with regard to weight management, physical activity and diet according to the model of Prochaska, and behavioral determinants such as attitude, social support, self efficacy with regard to weight management, physical activity and nutrition. Possible confounders that are assessed include: age, age at menarche, education, ethnicity, marital status, smoking behaviour and individual health status, including complications during pregnancy, the duration of labour and possible complications during or after delivery.

### Process evaluation

After the intervention, a process evaluation will be performed amongst participating midwives and respondents. In the process evaluation midwives will be asked under what conditions they would be willing to provide this type of intervention themselves. It will be assessed also whether and under what circumstances assistants, present in some practices, can execute the intervention program.

It is likely that practices who are interested in participating in a study about weight gain during pregnancy are practices that are already paying more attention to this subject more compared to other practices. In order to identify such differences between participating and non-participating practices, questions about the time spent by midwifes on subjects related to weight gain or lifestyle are incorporated.

Furthermore, 50 subjects will be asked how they experienced the intervention, and which factors influenced success or failure of the intervention. Also 50 women from the control group will be asked about the regular care, received from the midwives during pregnancy. The results of the process evaluation will be used to facilitate future implementation.

### Statistical analyses

Analyses will be performed to estimate the effect of the intervention on five domains: 1) total weight gain during pregnancy 2) the percentage of women gaining weight above the recommended IOM guidelines; 3) weight retention at six months and one year postpartum; 4) physical activity levels; and 5) behavioral determinants of weight management, physical activity and nutrition.

Longitudinal logistic regression analysis will be used for dichotomous outcome measures, and longitudinal linear regression analyses will be performed for all other outcome measures. In these analyses, the correlation between multiple measurements within one individual is taken into account. The regression coefficient reflects the average difference in the outcome variable between conditions. Possible confounders will be entered into the regression analyses. Confounding will be defined as a change in the regression coefficient of 10% or more. Possible effect modification will be studied as well, defined as a significant (p <0.10) interaction-term between group allocation and the variable concerned. Datawill be analysed according to the intention-to-treat principle and the per protocol principle.

## Discussion

The New Life(style) intervention program is designed to fill the gaps that currently exist for public health professionals with regard to providing guidance for pregnant women who have difficulties with controlling their weight gain during pregnancy. As a result of the close cooperation with the participating midwife practices in this study, we are able to reach a representative study population of women who are expecting their first child. Furthermore, the counselling sessions of participants in the intervention group and the measurements of all participants are performed in these practices, hereby minimising the burden for women who participate in the study. Because of the personal and repetitive character the New Life(style) program is expected to be a helpful tool to help pregnant women to gain weight within IOM-guideline limits.

Results of the current RCT will improve insight in determinants of weight gain during pregnancy, weight retention after childbirth and in the effectiveness of the intervention program. If the New Life(style) intervention program proves to be an effective tool, a future update of the Dutch and/or international guidelines of midwifery care with the current protocol will be needed. Because of the fact that the counselling sessions in this study are provided by members of the research team, implementation of the New Life(style) protocol in the daily practice of midwifes will then need to receive further attention.

## Competing interests

The author(s) declare that they have no competing interests.

## Authors' contributions

EA is responsible for the data collection, drafted the manuscript and developed the intervention protocol that is described. MvP originated the idea for the study, led on its design, and supervised the project. MvP and WvM were co-applicants on the successful funding proposal. CvdW participated in the recruitment of midwifery practices. All authors participated in discussing the design of the study and developing the research protocols. All authors read and corrected draft versions of the manuscript and approved of the final manuscript.

## Pre-publication history

The pre-publication history for this paper can be accessed here:



## Figures and Tables

**Table 1:** Content of individual counselling sessions

**Elements**	**C1**	**C2-4**	C5*
Introduction/general	X	X	X
weight gain during pregnancy	X		
weight gain chart	X	X	
feedback on weight gain	X	X	
relation of weight & lifestyle	X		
assessment of eating & PA	X	X**	X
feedback on eating & PA	X	X**	X
- review of – behavioral goals	X	X	X
recent experiences (labour, breastfeeding)			X
brochure	X		

* C5 is executed by phone

** optional elements
